# Prediction of electrical load demand using combined LHS with ANFIS

**DOI:** 10.1371/journal.pone.0325747

**Published:** 2025-06-10

**Authors:** Ahmed G. Ismail, Sayed H. A. Elbanna, Hassan S. Mohamed

**Affiliations:** 1 Sec. of Operation & Control, North Cairo Distribution Company, Ministry of Electricity & Energy, Cairo, Egypt; 2 Electrical Power and Machines Department. Faculty of Engineering, Al Azhar University, Cairo, Egypt; Vietnam Maritime University, VIET NAM

## Abstract

Enhancement prediction of load demand is crucial for effective energy management and resource allocation in modern power systems and especially in medical segment. Proposed method leverages strengths of ANFIS in learning complex nonlinear relationships inherent in load demand data. To evaluate the effectiveness of the proposed approach, researchers conducted hybrid methodology combine LHS with ANFIS, using actual load demand readings. Comparative analysis investigates performing various machine learning models, including Adaptive Neuro-Fuzzy Inference Systems (ANFIS) alone, and ANFIS combined with Latin Hypercube sampling (LHS), in predicting electrical load demand. The paper explores enhancing ANFIS through LHS compared with Monte Carlo (MC) method to improve predictive accuracy. It involves simulating energy demand patterns over 1000 iterations, using performance metrics through Mean Squared Error (MSE). The study shows superior predictive performance of ANFIS-LHS model, achieving higher accuracy and robustness in load demand prediction across different time horizons and scenarios. Thus, findings of this research contribute to advanced developments rather than previous research by introducing a combined predictive methodology that leverages LHS to ensure solving limitations of previous methods like structured, stratified sampling of input variables, reducing overfitting and enhancing adaptability to varying data sizes. Additionally, it incorporates sensitivity analysis and risk assessment, significantly improving predictive accuracy. Using Python and Simulink Matlab, Combined LHS with ANFIS showing accuracy of 96.42% improvement over the ANFIS model alone.

## 1. Introduction

Load demand (LD) problems in the medical section caused by over and no-load demand were crucial and repeated, such as failures, trips, voltage fluctuation causes voltage drop that affect service provider and hospitals [1]. Furthermore, depending on human monitoring and regular methods used in demand prediction causes faults lead to damage of network installations and even medical equipment, especially in neonate incubation and intensive care units. Moreover, responses in redistributing the loads on distribution network going to be un-accurate. Thus, causes several residual, secondary risks [2,3]. One of the most important problems that still exists and still affects the network is load transfer maneuvers on hybrid systems, which are still carried out manually, especially in distributed generation (DG) [4]. Load demand forecasting should respect risk factors, especially probabilities and impacts [5]. The accurate prediction of load demand is essential for ensuring reliability and efficiency of modern power systems [6,7]. However, traditional forecasting methods often struggle to capture intricate dynamics and uncertainties inherent in load demand data, leading to suboptimal decision-making and resource allocation. In addition, traditional load forecasting approaches, that rely on linear models, often cannot account for the complex and nonlinear relationships inherent in load demand data. These methods struggle to adapt to the rapid changes introduced by distributed generation and dynamic load transfers, causing delays in redistributing loads across the network and introducing residual risks [8]. Recent research highlights the need for more advanced, adaptive models that can handle the complexities of modern power systems, in high-stakes environments such as hospitals [9]. To address these challenges, this paper proposes a novel approach that integrates ANFIS with LHS to enhance load demand prediction accuracy. ANFIS is a hybrid model that combines the learning capabilities of neural networks with the interpretability of fuzzy logic, making it well suited for capturing the nonlinear and uncertain pattern characteristics of load demand [10,11]. By incorporating LHS, a stratified sampling method that ensures better coverage of the input space compared with random sampling methods such as MC, this approach optimizes the forecasting process [12]. Recent studies on load forecasting, such as those leveraging bidirectional LSTM networks, have demonstrated improvements in predictive accuracy by capturing temporal dependencies in load data [13]. However, the limitations of these models lie in their complexity and requirement for large datasets. In contrast, combining ANFIS with LHS not only provides robust predictions with less data but also offers a more interpretable model, making it ideal for applications in healthcare and other critical infrastructure sectors. This paper studied contributes to the field by demonstrating that integrating ANFIS with LHS outperforms traditional models such as LSTM and ANN, in scenarios with noisy or incomplete data. In summary, this study presents an advanced forecasting framework that leverages the adaptability of ANFIS and sampling efficiency of LHS, enabling more accurate, reliable, and interpretable load demand predictions. This innovative approach holds promise for improving decision-making and resource allocation in power systems, in high-risk environments such as hospitals [14,15]. Electrical load demand prediction is critical for efficient energy management, particularly in healthcare facilities where accuracy is essential for life-saving operations. However, existing methods often struggle with challenges such as overfitting, inefficiency in handling high-dimensional input spaces, and limited adaptability to varying data sizes. To address these limitations, this study proposes a novel hybrid methodology that integrates LHS with ANFIS to achieve high accuracy in LD prediction with minimal computational time and iterations, while addressing potential interpretability limitations of ANFIS. The methodology comprises four stages beginning with data collection (Historical electrical LD data, including variables such as weather conditions, energy consumption, grid infrastructure status, and market dynamics, that is clarified and mentioned in methodology section. Thus, preprocessing data by cleaning outliers, handling missing values, and normalizing features for consistent scaling. Second stage LHS Generation through input parameters defined, ranges determined, and divided into equal intervals. third stage is ANFIS Development model is validated, tested, and refined to achieve optimal performance. Last stage is optimization through LHS generated samples used as inputs for ANFIS, iteratively optimizing model to achieve best prediction accuracy. Using Python and MATLAB simulation discussed in details in case studies, three standalone methods and the fourth is the research study combined LHS with ANFIS.

The originality of this work lies in its ability to ensure structured, stratified sampling of input variables, significantly reducing overfitting and enhancing adaptability through using LHS, while ANFIS captures complex nonlinear relationships inherent in load demand data. The primary research question driving this study is: How can the accuracy, robustness, and computational efficiency of electrical LD forecasting in healthcare facilities be enhanced? Additionally, the study investigates the applicability of strategies to mitigate overfitting, conduct sensitivity analysis, and perform risk assessment to quantify uncertainty, while also examining the potential impact of these approaches. The organization of this paper is outlined as follows: Section 1 presents the background and the existing state of research in this field. Section 2 provides an overview of the literature review. Section 3 details the proposed methodology, while Section 4 explains the experimental setup. Section 5 discusses the analysis of the results obtained. Section 6 delves into further analysis and discussion of these findings. Finally, Section 7 concludes the paper and outlines future research directions.

## 2. Literature review

The field of electrical load demand forecasting has witnessed applying various machine learning techniques, each offering distinct strengths and weaknesses depending on the data and the forecasting requirements. This review outlines some of the most employed models, highlighting their advantages, limitations as indicated in Table 1, and relevance to energy forecasting.

**Table 1:** Negatives in previous used techniques.

Techniques	Negatives
ANNs (incl. MLPs)	Computational Cost, Black Box Problem [16,17]. Data Dependency [18].
SVMs with RBF kernels	Computationally expensive.
Monte Carlo Simulation	Computational Intensity, Accuracy Relies on Assumptions [19].
LSTMs	Vanishing Gradient Problem, require a substantial amount of historical data, computationally expensive [20].
GPR	More complex to implement compared to SVMs [21].
Ensemble Learning	Complexity Management [22].
Deep Reinforcement Learning	Immaturity of the Technique, High Computational Requirements [23].
ANFIS	Limited interpretability [24].

Smart grid development has drawn noteworthy attention for exploration and advancement in the most recent decade [25,26]. Forecasting electrical load demand is a process that aims predicting future electrical load of system [27]. It is an important tool for many power system operations, included but not limited unit duty planning, power purchase scheduling, and infrastructure development [28]. Inaccurate forecasting may cause an extra cost on asset operation and system support. On the other hand, inadequate forecasting may cause an unsatisfied client due to an electric power deficiency [29]. Previous methodologies for load demand prediction have typically included statistical techniques, such as time series analysis, regression models, and artificial neural networks (ANNs) [30]. Time series analysis methods, including autoregressive integrated moving average (ARIMA) models, have been widely used for capturing temporal patterns and trends in load data [31]. Regression models, such as linear regression and multiple regression, have been employed to identify and quantify the relationships between load demand and various influencing factors, such as temperature, wind speed, consumption and economic indicators [32].

In recent years, there has been growing interest in leveraging advanced computational techniques to enhance the accuracy and efficiency of predictive modeling in various domains.

ANNs used a machine learning model known for their capacity to approximate non-linear functions and handle complex relationships within data [33]. As McCulloch and Pitts (1943) proposed, ANNs have since been applied to various forecasting tasks, including load demand prediction [34]. Their versatility and ability to model non-linear dependencies has made ANNs a popular choice [35]. Nevertheless, ANNs tend to require large amounts of labelled data to generalize effectively, and they are prone to overfitting when trained on smaller datasets. Furthermore, ANNs, like LSTMs, often lack interpretability, limiting their applicability in scenarios where model transparency is vital [36].

Multilayer Perceptron (MLP) is a specific class of feed-forward ANN, consisting of multiple layers of neurons capable of approximating any continuous function. As highlighted by Rumelhart et al. (1986), MLPs form the foundation of deep learning and are highly versatile for both classification and regression tasks [37]. MLPs excel in handling non-linear data but suffer from vanishing gradient issues in deep architectures, which limit their effectiveness in deeper networks [38]. Additionally, MLPs require significant computational resources and time for training, especially for larger datasets.

Radial Basis Function (RBF) networks, introduced by Broomhead and Lowe (1988), are a type of artificial neural network that uses radial basis functions as activation functions [39]. RBF networks are effective for interpolation tasks and are used in function approximation and regression problems [40]. However, RBF networks tend to struggle with high-dimensional data because of the curse of dimensionality [41]. Additionally, they offer only local generalization, limiting their ability to make accurate predictions outside the range of the training data.

Multilayer perceptron (MLP) neural networks and radial basis function (RBF) networks are examples of ANN architectures commonly applied in load demand prediction tasks [42,43].

Support Vector Machines (SVM), introduced by Cortes and Vapnik (1995), are a powerful supervised learning technique commonly used for classification and regression tasks [44]. SVMs perform well in high-dimensional spaces and are robust against overfitting, especially when the dataset is limited [45]. The strength of SVMs lies in their ability to maximize the margin between classes, enhancing their predictive performance. However, SVMs can be computationally expensive, particularly when applied to large datasets. The choice of kernel functions is also critical, as improper kernel selection can negatively impact performance.

Support Vector Machines (SVM) with RBF kernels was a popular choice for electrical load demand prediction. Hence, they can effectively capture non-linear relationships in factors affecting load included but not limited (weather, grid infrastructure) [46].

MC methods are commonly used for uncertainty quantification in forecasting models. Metropolis and Ulam (1949) demonstrated the value of MC simulations in estimating the probability of different outcomes under conditions of uncertainty [47]. However, the major limitation of MC simulations lies in their computational intensity, requiring many iterations to produce reliable results [48]. In addition to, the randomness inherent in the method can lead to variable outcomes unless sufficient iterations are performed [49].

In load demand prediction, MC simulation is often used to generate multiple scenarios of future load demand based on probabilistic distributions of historical data [50]. It provides a comprehensive understanding of the range of possible outcomes and associated uncertainties [51].

While these traditional methodologies have shown some success in load demand forecasting, they often face challenges in accurately capturing the nonlinear and dynamic nature of load data, as well as handling uncertainties and variations in external factors.

Long Short-Term Memory (LSTM), a specialized recurrent neural network (RNN) model, was designed to overcome the vanishing gradient problem that hampers traditional RNNs [52,53]. LSTMs excel in capturing long-term dependencies in sequential data, making them well-suited for time-series prediction, such as electrical load demand forecasting [54]. Hochreiter and Schmid Huber (1997) highlighted the advantages of LSTM in processing sequences of data with long-range dependencies. However, LSTMs are computationally expensive and require large datasets for training, which can be a limitation when data availability is constrained [55]. Additionally, LSTMs function as “black-box” models, offering little interpretability, which can be a drawback when transparency is required in decision-making [56,57].

Gaussian Process Regression (GPR) is a Bayesian non-parametric method that predicts distributions over functions and is useful for capturing uncertainties in regression problems [58]. As highlighted by Rasmussen and Williams (2006), GPR provides confidence intervals along with predictions, making it valuable in applications where uncertainty quantification is critical [59,60]. However, GPR does not scale well with large datasets because of its cubic time complexity, and its performance depends highly on the appropriate selection of kernel functions [61,62].

Ensemble learning techniques combine multiple models to improve predictive accuracy. Breiman (1996) introduced bagging as one of the earliest ensemble techniques, which improves model performance by reducing variance and bias [63]. Ensemble methods are highly effective in improving generalization and robustness, making them suitable for complex forecasting tasks like load demand prediction [64]. However, ensemble learning can be computationally expensive and often lacks interpretability, as the combined effect of multiple models is harder to explain [65].

Deep Deep Reinforcement Learning (DRL) combines reinforcement learning with deep learning techniques, allowing agents to learn decision-making policies through interaction with an environment. Mnih et al. (2015) demonstrated the effectiveness of DRL in achieving human-level control in complex environments. Because DRL can autonomously learn policies, it is suitable for dynamic load management in energy systems [66]. However, DRL suffers from sample inefficiency, requiring numerous interactions to converge on an optimal policy [67]. Moreover, balancing exploration and exploitation can be challenging, often resulting in suboptimal learning.

The two-stage day-ahead multi-step wind power prediction scheme described addresses limitations of Numerical Weather Prediction data in high penetration wind power grid connections, which are critical for the safe and stable operation of power systems. This method, leverages temporal interactions and a deep decomposition module to enhance forecasting accuracy, contrasting with paper approach that combines LHS with ANFIS to optimize input sampling and model adaptability for electrical load demand forecasting. Experimental analyses from wind farms demonstrate efficacy of this multi-step scheme, showing superior performance to other evaluated methods [68]. In comparison, LHS with ANFIS methodology focuses not just on improving accuracy but also on ensuring robustness and interpretability in load demand predictions within healthcare facilities.

In addressing challenges of volatility and unpredictability of wind power, study proposes a dual numerical weather prediction (NWP) wind speed correction method that utilizes trend fusion and fluctuation clustering to enhance accuracy of short-term wind power prediction. This approach distinctly contrasts with our combined LHS with ANFIS methodology, which focuses on optimizing input sampling and enhancing adaptability across variable load demands in healthcare facilities. Specifically, dual NWP method, demonstrated in wind farms, incorporates Complete Ensemble Empirical Mode Decomposition with Adaptive Noise (CEEMDAN) and an Attention-GRU model to refine NWP data, successfully improving prediction accuracy by 10% and 13.1% [69].

The ANFIS model, introduced by Jang (1993), The primary strength of ANFIS lies in its ability to model non-linear relationships through fuzzy rules while maintaining interpretability—an essential factor in energy load forecasting [70]. This interpretability enables operators to comprehend and adjust fuzzy rules based on domain knowledge, making ANFIS surprisingly valuable in environments requiring transparency, such as energy management systems.

ANFIS is a hybrid intelligent system that combines adaptive capabilities of neural networks with linguistic representation of fuzzy logic [71]. It learns from data to model complex, nonlinear relationships between input and output variables [10]. The ability to handle uncertainty and nonlinearities in data has led to widespread use of ANFIS in load demand prediction [72]. Although, ANFIS faces a challenge of limited interpretability refers to difficulty of understanding how a model arrives its predictions (Complex Algorithms, Non-Linear Relationships, Black Box Effect) [73]. LHS is a stratified sampling method that ensures efficient exploration of input space by dividing it into probable intervals [74]. Unlike simple random sampling, LHS ensures a more uniform coverage of input space, leading to better representation of variability in input variables. Combining LHS with other modeling techniques, such as ANFIS, can improve prediction accuracy and robustness in load demand prediction [75].

This motivates exploration of advanced techniques, such as integration of ANFIS with LHS, to improve accuracy and reliability of load demand predictions. Among these techniques, LHS and ANFIS have emerged as powerful tools for optimizing sampling strategies and improving performance of predictive models.

Each of the models reviewed has unique strengths and weaknesses, with their applicability depending on the specific requirements of the forecasting task. LSTM and ANN models excel in handling large datasets and non-linear relationships, they often lack interpretability, a key factor in energy forecasting. ANFIS provides a balance between accuracy and interpretability but struggles with high-dimensional or temporal data [76]. Ensemble learning and MC methods offer robustness and uncertainty modelling but come with increased computational costs. Choosing the right model for load demand prediction in smart grids, therefore, depends on factors such as obtaining data, the need for interpretability, and computational resources.

The previous literature reveals that ANFIS shows a balanced performance across key criteria, such as robustness, accuracy, uncertainty handling, and interpretability [77]. ANFIS provides a well-rounded approach, making it a strong candidate for protection systems, where interpretability and uncertainty handling are important.

This research builds upon innovative leverage by integrating LHS with ANFIS, leveraging strengths of both techniques. LHS optimizes input sampling, ensuring a well-distributed dataset for training, while ANFIS enhances predictive accuracy by modeling complex relationships in LD data. The proposed LHS with ANFIS hybrid model offers a novel solution by achieving high forecasting accuracy with 96.42% improvement over standalone ANFIS, demonstrating superior robustness, efficiency, and adaptability. Unlike previous models, this study also incorporates LHS driven sensitivity analysis, providing deeper insights into parameter influence and further enhancing model performance. These contributions position LHS with ANFIS as a practical and efficient forecasting tool for energy management, particularly in critical infrastructures such as hospitals, where accurate load prediction is essential for operational stability.

## 3. Methodology

The proposed method is combined LHS with ANFIS to reach the best accuracy in load demand prediction with least time and minimum iterations preventing any limited interpretability in ANFIS. Moreover, this prediction helps in:

1- Establish a strong energy management system (EMS).
2- Help in re-arrange either the loads or the energy resources in Distributed generation.
3- Insure efficient demand response.
4- Accurate schedule for maintenance of electrical grid installations.
5- Grantee electrical grid which prevents blackout caused by trips or failures of electrical installations due to load fluctuations or peak periods.
6- Save time and cost.

Methodology steps include:

- Collecting Data and Preprocessing.
- Generate LHS.
- Generate ANFIS.
- Optimizing results using combined LHS with ANFIS.

### 3.1 Sensitivity analysis

Allows to better understand reliability and applicability of model, which is crucial for validating;

1. Identifying key parameters or inputs of model. Systematically varying these parameters finding how changes influence model’s outputs.
2. Determining which parameters have most significant effect on results. might involve quantitative measures to find which parameters, when altered, lead to substantial changes in outcomes as a result assess impact.
3. Sensitivity analysis helps in understanding the uncertainty in predictions made by model. Highlights which assumptions are critical and which are less important.
4. Analyzing how robust the model outcomes are to variations in input parameters, conclusion about model’s appropriateness for specific context it is meant to address. In case of small changes in inputs lead to large changes in outputs, model may not be robust enough for practical use. However, if the model maintains predictive accuracy despite moderate fluctuations in inputs, it demonstrates strong adaptability and reliability for practical use.
5. Finally, documentation and reporting findings. Indicating which parameters had been analyzed, how changes were implemented, and what implications are for suitability of model.

#### 3.1.1 Outcomes.

- The model exhibited consistent performance across a wide range of parameter variations (±20%), with MSE fluctuations remaining within an acceptable range.
- Grid Load and Energy Consumption had the most significant effect on MSE, variations in LD and infrastructure status directly influence prediction accuracy.
- Temperature and Wind Speed exhibited a moderate effect, reinforcing their relevance but highlighting model’s ability to handle environmental variations effectively.
- Electricity Price had least impact, indicating that while market dynamics influence load demand, they do not heavily affect short-term forecasting accuracy.

As a result, from Table 2. provided inputs are highly suitable for combining ANFIS with LHS. This combination can effectively model nonlinear relationships in data, perform sensitivity analysis, and quantify uncertainty. It is particularly useful for understanding how percentage changes impact key parameters like Temperature, Wind Speed, Energy Consumption, Grid Load, and Electricity Price. This approach provides valuable insights for system optimization, decision-making, and predictive modeling.

**Table 2:** Impact of sensitivity analysis on model performance.

Variable/Variation%	−20%	−15%	−11%	−6%	−2%	2%	6%	11%	15%	20%
**Temperature**	40.76572	47.15391	31.71659	38.33709	32.02765	48.66938	34.89367	48.78076	45.49189	42.79675
**Wind Speed**	48.3641	32.07557	49.2344	36.42024	48.67465	34.92533	45.13857	49.20881	35.76391	43.9416
**Energy Consumption**	34.12629	31.82318	39.52617	47.89879	32.73369	41.50343	33.85545	38.03557	32.19513	33.85029
**Grid Load**	45.67387	33.06291	47.26752	38.7591	33.01315	37.55563	33.52142	32.50948	45.10569	47.36728
**Electricity Price**	31.56361	38.39869	49.1278	37.34992	41.68412	45.60973	48.36121	32.99403	30.21498	46.72395

### 3.2 Collecting data and preprocessing

Gathering historical data of electrical load demand, including variables such as weather conditions, energy consumption, grid infrastructure status, market dynamics and number of iterations.

Pre-process the data by cleaning outliers, handling missing values, and normalizing the features to ensure consistent scaling.

### 3.3 The most important conditions

1. The data used for prediction models representative of actual electrical load demand, including seasonal variations, day and night peak demand periods, and potential anomalies. However, data used in prediction models is carefully chosen to reflect real-world electricity use, including how demand changes with seasons, difference between day-time and night-time usage, and unexpected changes in energy consumption. This ensures that the model can accurately predict future electricity needs, helping power systems operate smoothly, avoid shortages or overloads, and improve overall energy management. By considering these factors, model supports better decision-making for balancing electricity supply and demand, ensuring stable and efficient energy distribution.
2. All data of healthcare facilities, which have unique and dynamic energy consumption patterns due to their 24/7 operational nature and critical infrastructure.
3. The methodologies applied (machine learning algorithms, sensitivity analysis, and risk assessment) are feasible and computationally viable within constraints of available resources and time.

### 3.4 Study is bounded by the following limitations

1. Study need more actual external factors such as grid reliability, policy changes, or extreme weather events are either controlled or accounted for in the uncertainty quantification process.
2. Applicability of findings may be limited to healthcare facilities with similar operational characteristics, and generalization to other sectors (commercial or residential buildings) that require further investigation.
3. One of most crucial boundaries that is often omitted in load demand prediction models is load growth, which is already added in the medical section.

### 3.5 Approaches to generate LHS sample

1- Define input parameters, from collected data.
2- Determine range of each parameter.
3- Dividing range into equal intervals for each input variable and one sample is selected randomly from each interval. This process is repeated until the desired number of samples is generated.
4- Generate random values within each interval to get variant values and combine it for samples.
5- Use the samples with ANFIS until getting the optimum model.

The samples generated by LHS are used as the initial population for optimization algorithm, algorithm run multiple times to find the optimal solution.

### 3.6 Approaches to generate ANFIS

ANFIS combines the strengths of neural networks and fuzzy logic.

1. Define Fuzzy Inference System (FIS) Structure
2. Network Initialization.
3. Learning Algorithm.
  - Hybrid Learning.
  - Gradient Descent.
4. Training.
5. Validation and Testing.
6. Model Refinement (Optional).

## 4. Case study

The selected electrical distribution grid serves hospital comprises four buildings, first building is outpatient clinic, second is an administrative building, housing is third building. However, the fourth building is the most important as it includes the operation rooms, intensive care, pediatric incubation and emergency room. Distribution network may find too much critical risks as the problem is that even the flicker is not accepted in this building. Moreover, ventilations, and elevators even water pumps and other several issues, may be caused in case of electrical fluctuates. This electrical infrastructure supplied by 11 kV. Two feeders from different substations and an energy storage consist of (Lithium-Ion Batteries).

### 4.1 Time

It is required to energize in-patient building in peak periods or sudden blackouts instead of grid power, as peak period is going to be from 6 pm to 10 pm with one more hour to be standing for five hours. Charging time is about five to six hours.

### 4.2 Time calculation (charging and discharging)

- Time required for charging or discharging a battery is determined based on the energy requirements and efficiency of the battery system. Formula for calculating discharge time is:

$$Discharge\;Time=\frac{Energy\;Needed}{Power\;Load}\;\times \;Efficiency$$ (1)

Efficiency factor accounts for energy losses during conversion and can vary depending on the type of battery. Lithium-ion batteries have an efficiency range of 85–95%.

### 4.3 Capacity calculation

To calculate the energy capacity of the battery required to supply a specific load, the formula used is:


$$Energy\;Capacity\;(kWh)=\frac{Load\;(kW)\times \;Time\;(hours)}{Battery\;Efficiency}$$ (2)

### 4.4 Capacity

- For a 1 MW load over 5 hours with 90% efficiency, this becomes:

This formula is used in energy storage system design and sizing calculations [78].


$$Energy\;Capacity\;(kWh)=\frac{1000\;(kW)\times \;5\;(hours)}{0.9}=5.56\;mWh$$ (3)

### 4.5 Battery size calculation


$$Battery\;Size\;(Ah)=\frac{Energy\;Capacity\;(kWh)}{Nominal\;Voltage\;(V)}$$ (4)

- For a 5.56 mWh energy requirement and a nominal voltage of 3.7V, the calculation is:

$$Battery\;Size\;(Ah)=\frac{5,560\;kWh}{3.7\;V}=1,502,702Ah$$ (5)

- This calculation ensures the battery can deliver energy over the specified time period [79].
  - Power rating of batteries calculated by dividing total power by discharge time: 1 mW/ 5 hours = 200 kW
  - Therefore, the required size of lithium-ion batteries for a 1 mW load for 5 hours is 1,502,702 Ah with a power rating of 200 kW.

### 4.6 Size

- Nine battery racks are needed to get to 1 megawatt.

#### 4.6.1 Basic information (for selected feeders).

**Fig 1:**
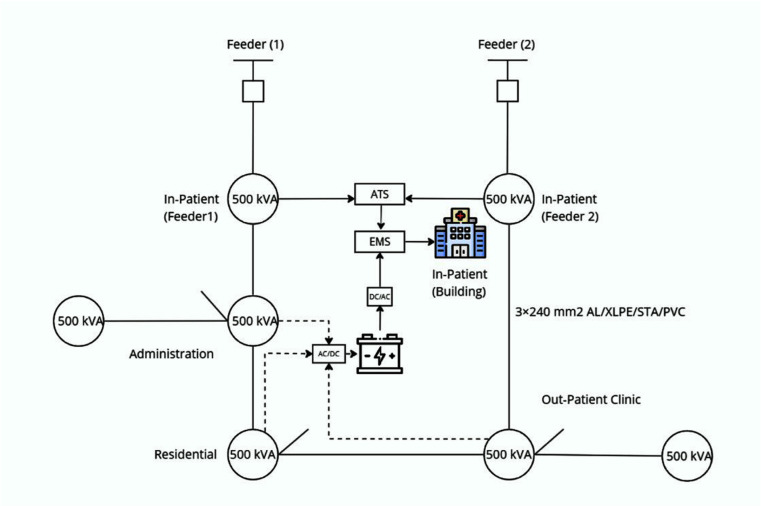
Distribution grid.

Fig 1 shows distribution grid installations that comprise:

- Cables are typical in a network as 3 × 240 mm2 AL/XLPE/STA/PVC.
- Five power transformers their capacity and cooling type are 500 kVA, 50 Hz, Dyn11, ONAN, 11/0.4 kV.
- Energy storage (Lithium-Ion Batteries);

Predicting load demand and redistribute to avoid risk of any failures because of peak overload.

method uses the collected data as it is shown in Table 3:

**Table 3:** Parameters of influence factors.

Category	Parameter	Minimum	Maximum	Unit
Weather Conditions	Temperature	−10	40	°C
	Wind Speed	0	20	m/s
Energy Consumption Patterns	Consumption	100	2000	kWh
Grid Infrastructure Status	Grid Load	0	5000	kW
Market Dynamics	Electricity Price	0.1	0.3	$/kWh
Simulation	Number of Iterations	1000		Iterations

**Fig 2:**
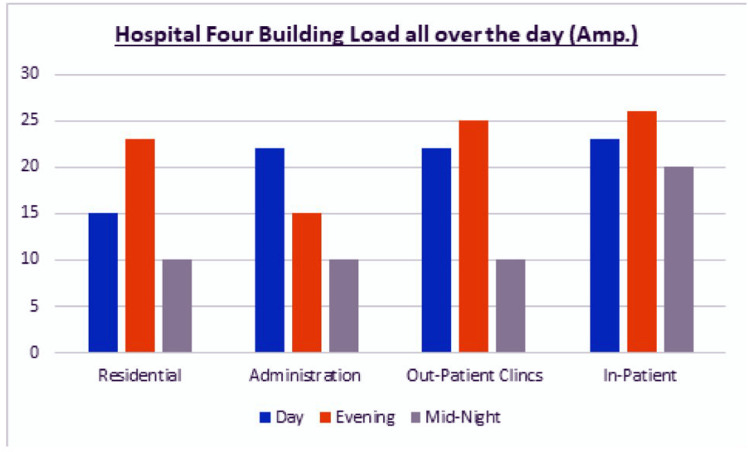
Load profile for hospital buildings before using EMS/ energy storage.

Fig 2 indicates hospital four buildings load profile on whole day time. Analysis clarifies that residential, administration and outpatient clinics buildings are stable rather than inpatient building loads which is fluctuating severely causing inverse various peaks.

**Fig 3:**
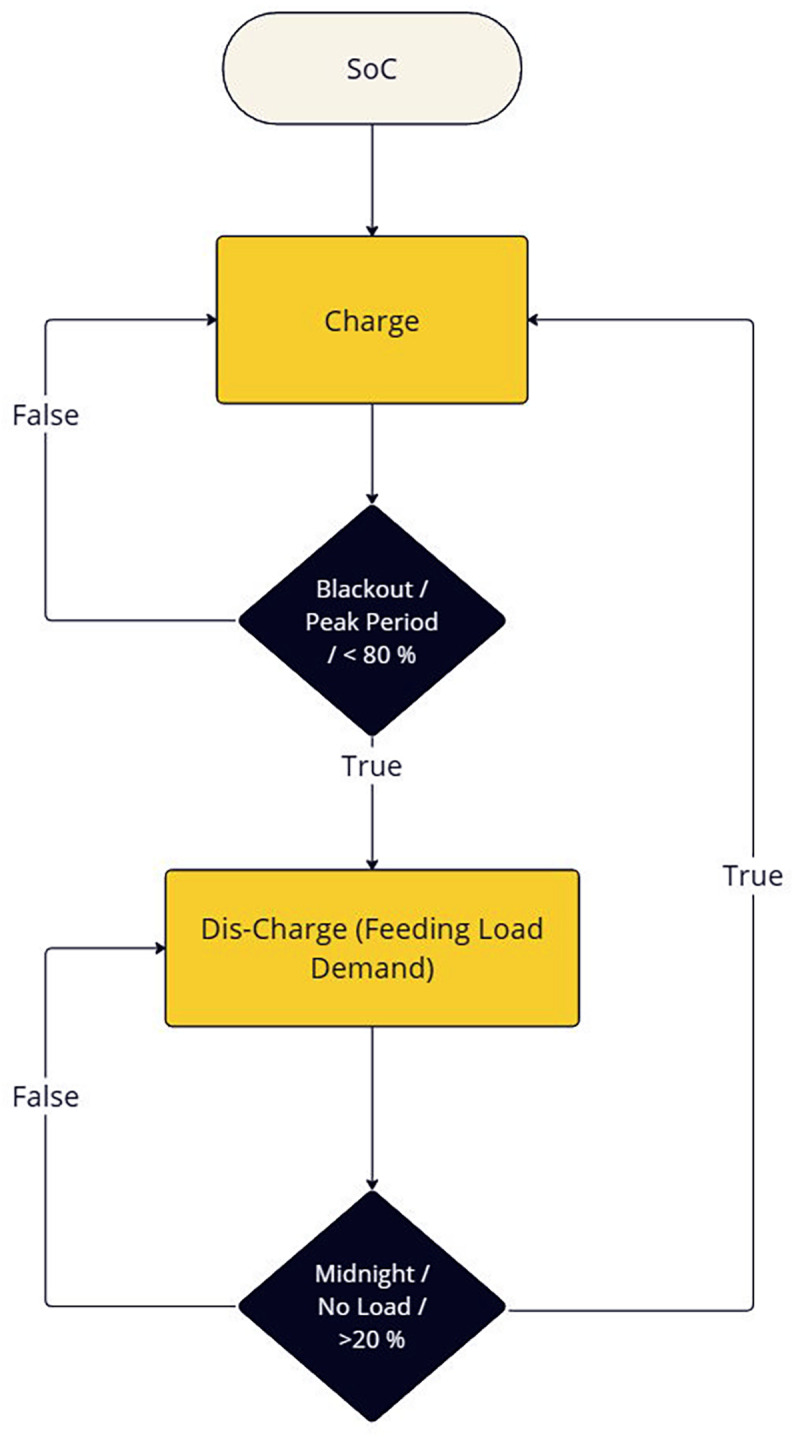
EMS controlling SoC.

Flowchart in Fig 3 illustrates the decision-making process for a battery system based on its State of Charge (SoC). The system charges under normal conditions but discharges to meet load demand during blackout or peak periods when the SoC is below 80%. Discharging continues until conditions like midnight or no load with SoC above 20% are met, optimizing energy usage and storage.

### 4.7 Methodology 1

Using MC analysis, provides a single point estimate, neglecting inherent uncertainty in future predictions.

Approaches to generate MC analysis

- Identify key factors influencing load demand.
- Assign probability distributions to each factor based on historical data.
- Random number generator to sample values from each factor probability distribution. creating a single simulated scenario for load demand.
- Repeating step 3 a thousand of times to generate numerous simulated scenarios. Analysing the result distribution of forecasted demands to understand the range of possibilities and predict.

### 4.8 Methodology 2

Using ANFIS in predicting electrical load demand. Approaches to generate ANFIS.

- Data collection of previous load demand and relevant influencing factors weather conditions, energy consumption patterns, market dynamics, and grid infrastructure status.
- Preparing the data by handling missing values and outliers. Feature scaling was necessary for numerical stability.
- ANFIS model design and define the ANFIS architecture with input and output layers.
- Training model using historical data in the training process adjusts parameters of membership functions and neural network to minimize errors between predicted and actual load demand.
- Validation and testing, evaluate the model performance on unseen data to assess its generalizability. Common metrics include Mean Squared Error (MSE) and Mean Absolute Percentage Error (MAPE).
- Once validated, a trained ANFIS model used to forecast future load demand based on new input values.

**Fig 4:**
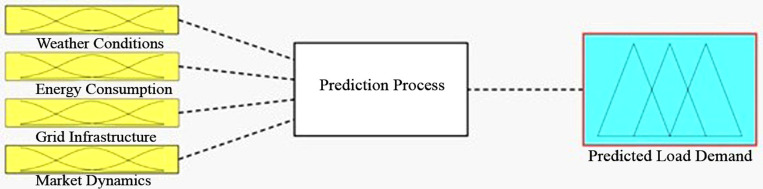
ANFIS model for the four parameters.

Fig 4 display stage of utilizing ANFIS model demonstrates the four parameters (weather conditions, energy consumption, grid infrastructure, market dynamics) as an input. Consequently, ANFIS control load flow on different sources (grid, energy storage and diesel generator). However, a diesel generator was not selected as a solution. Hence, the system charges power storage from the grid at midnight and discharge at peak periods.

**Fig 5:**
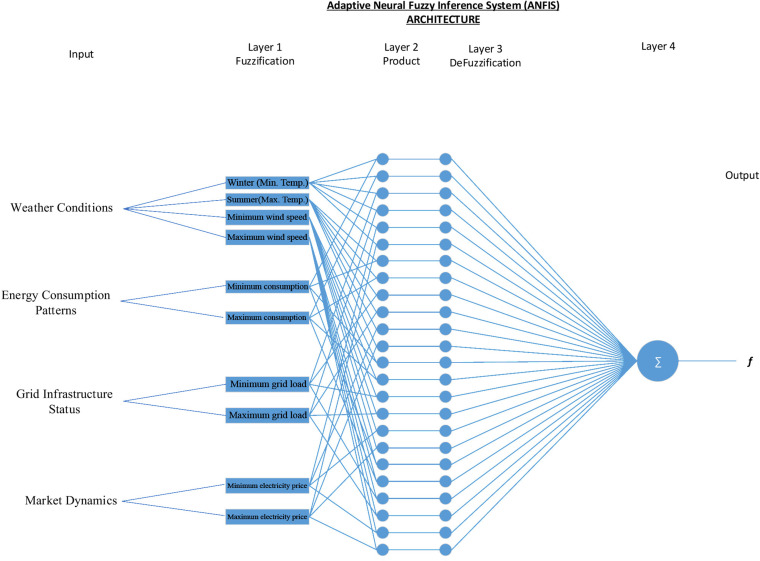
ANFIS structure.

Structure details of utilizing ANFIS model using information of four parameters as an input illustrated in Fig 5. Therefore, using ANFIS to control load flow between various sources (grid, energy storage, and diesel generator) allows for the selection of the best resource.

### 4.9 Methodology 3

Using LHS Unlike ANFIS and MC simulations, LHS plays a crucial role in forecasting for load demand.

- Divide distribution of each factor probability distribution is divided into equally probable intervals (strata).
- Random Sampling within srata, each factor of single value is randomly selected from each stratum, ensuring all regions of distribution have been represented.
- Combine Samples, randomly selected values from each factor strata are combined to form single simulated scenario for load demand.
- Predicting, once simulated scenario formed for load demand.

**Fig 6:**
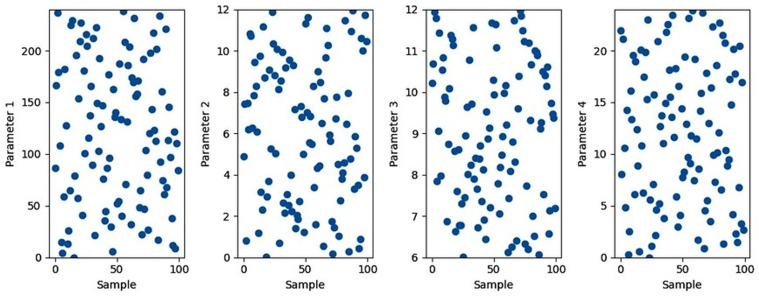
LHS model for the four parameters.

Fig 6 reveals scatter diagram of LHS for the four parameters (Weather Conditions, Energy Consumption, Grid Infrastructure, Market Dynamics). Indicating last approach in LHS which is random values. Thus, LHS generate samples to be used as inputs for ANFIS to simulate a model help in training ANFIS to achieve optimum one.

### 4.10 Methodology 4

Using combined LHS with ANFIS,

1. Compiling previous load demand data and pertinent influencing factors.
2. Data Preprocessing.
3. Create an ANFIS architecture with input and output layers and define the ANFIS model.
4. Use LHS to generate a set of input values that represent wide range of possible scenarios based on probability distributions of influencing factors.
5. Utilize LHS-generated input data and associated load demand values to train the ANFIS model.
6. Validation, testing, and performance evaluation of ANFIS model on unknown data in order to determine its generalization ability.
7. Once validated, use a trained ANFIS model to forecast future load demand based on new input values.

- LHS focuses on dividing the probability distribution range of each input factor (weather, energy consumption patterns, market dynamics and grid infrastructure status) into equally probable intervals (strata).

$x_{i}$ is i^th^ input factor (weather, energy consumption patterns, market dynamics and grid Infrastructure Status).

P($x_{i}$) is probability distribution function of $x_{i}$.

process involves:

1. Discretizing the Distribution by dividing the range of P($x_{i}$) into N sub-intervals (strata) with equal probability$\frac{1}{N}$.
2. Width ($\Delta_{i}$) of each sub-interval for factor $x_{i}$ using

$$\Delta_{i}=\frac{1}{N}$$ (6)

1. Random Sampling within Strata, for each factor (i) randomly sample one value (x_ij_) from each stratum j (1 to N). This ensures all areas of the distribution have a chance of being represented.

Data preprocessing and data splitting,

training set: D_train_, validation set: D_val_,testing set:D_test_.

ANFIS Model Design, Intialize ANFIS Parameters, Member ship functions MF_i_ (X) input variables X. Rules(R_j_).

Construct ANFIS Output;

Output of Rule_j_ for input X_i_: O_j_ = MF_i_(X) ⋅ Rule Weight_ij_

Overall ANFIS Output: O = ∑_j_O_j_

Training:


$$E_{train}=\frac{1}{D_{train}}\sum_{i=1}^{|D_{train}|\;}\;{\left(y_{i}+O_{i}\right)}^{2}$$ (7)

Where:

- $E_{train}$: Training error or the mean squared error (MSE) calculated over the training set.
- $D_{train}$: Training dataset, which is the set of all training samples.
- $y_{i}$: Actual target output (ground truth) for the iii-th training sample.
- $O_{i}$: Predicted output from the ANFIS model for the iii-th training sample.
- $|D_{train}|$: The number of training samples.

Validation:


$$E_{val}=\frac{1}{D_{val}}\sum_{i=1}^{|D_{val}|\;}\;{\left(y_{i}+O_{i}\right)}^{2}$$ (8)

Where:

- $E_{val}$: Validation error or the MSE calculated over the validation set.
- $D_{val}$: Validation dataset, which is the set of all validation samples.
- $y_{i}$: Actual target output (ground truth) for the iii-th validation sample.
- $O_{i}$: Predicted output from the ANFIS model for the iii-th validation sample.
- $|D_{val}|$: The number of validation samples.

Testing:


$$E_{test}=\frac{1}{D_{test}}\sum_{i=1}^{|D_{test}|\;}\;{\left(y_{i}+O_{i}\right)}^{2}$$ (9)

Where:

- $E_{test}$: Testing error or the MSE calculated over the test set.
- $D_{test}$: Testing dataset, which is the set of all testing samples.
- $y_{i}$: Actual target output (ground truth) for the iii-th test sample.
- $O_{i}$: Predicted output from the ANFIS model for the iii-th test sample.
- $|D_{test}|$: The number of testing samples.

## 5. Results

Therefore, combined predictions can be considered as best choice.

**Fig 7:**
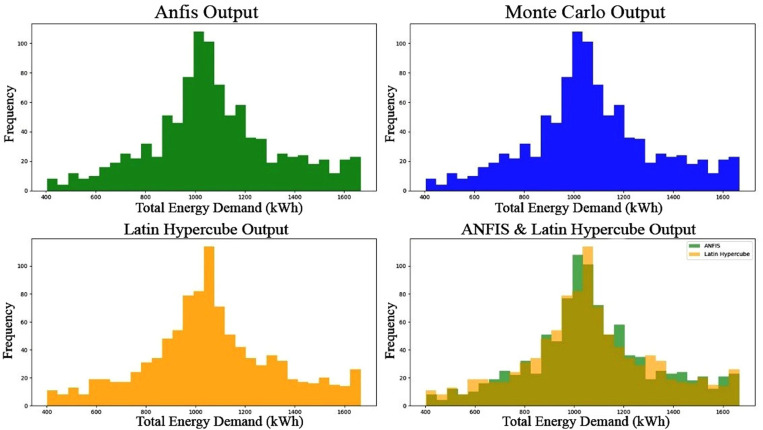
Total energy demand for the four scenarios.

Fig 7 reveals analysis compared performance of individual prediction methods (MC, LHS, ANFIS) and a combined prediction approach for LHS with ANFIS to predict energy demand. Combined predictions offer several key benefits:

- Reliable average demand from combined approach (1055.70 kWh) closely aligns with individual methods, showing a robust and accurate representation of average energy consumption.
- Conservative maximum demand estimates lower than individual methods, combined prediction’s maximum demand (1634.53 kWh) provides a more cautious estimate. This can be beneficial for planning purposes, as it helps avoid underestimating peak energy requirements.
- Conservative minimum demand estimates of combined prediction (446.93 kWh) is higher than individual methods, offering a more conservative estimate. This can be advantageous as it ensures sufficient capacity to meet even lower-than-expected demand scenarios.

**Fig 8:**
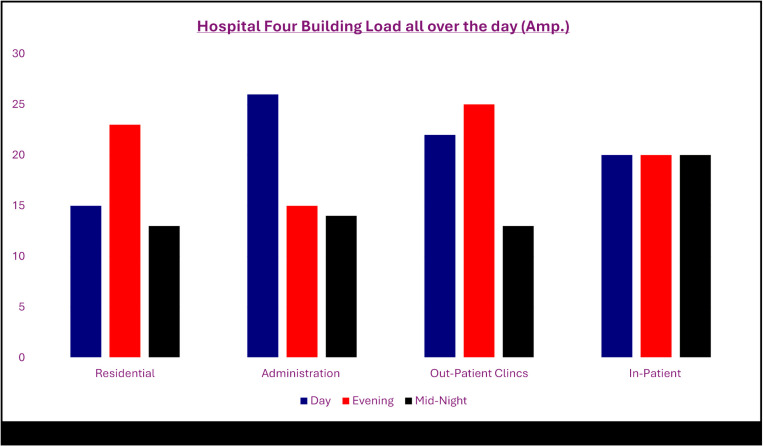
After redistributed load using EMS/ energy storage predicted by LHS with ANFIS.

Fig 8 proof success of utilizing LHS combined with ANFIS model smoothing load demand of in-patient building using EMS as proactive action to avoid blackouts or overload compared with Fig 2 which reveals the non-balanced loads.

**Fig 9:**
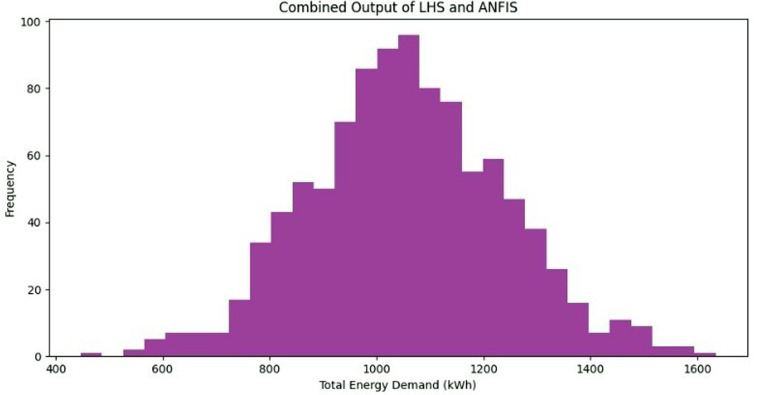
Total energy demand for the combined scenario.

Based on the analysis results overall indication, Fig 9 shows that combined predictions provide accurate prediction of energy demand.

**Fig 10:**
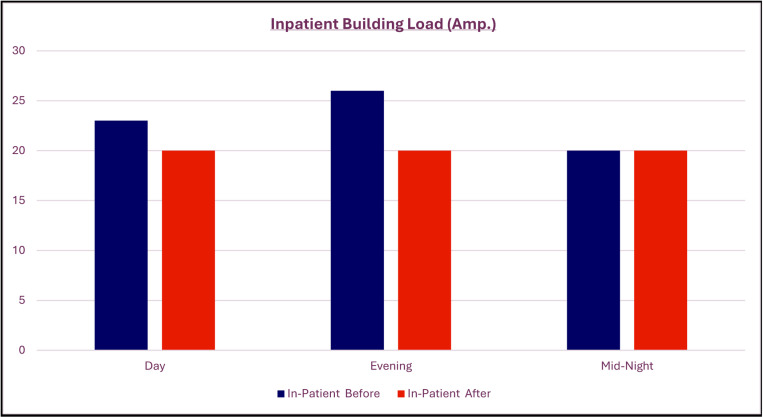
Results of demand response before and after using combined LHS with ANFIS.

As a result, combined LHS with ANFIS achieving in deciding a suitable source in optimum time, avoiding peak periods and side effects on network installations as per shown in Fig 10.

Table 4 shows comparison of energy demand estimated from different methods. Combined predictions provide a reliable estimate of energy demand.

**Table 4:** Four scenarios results.

Metric	Actual	Combined	MC	LHS	ANFIS
**Average Demand in kWh**	1050	1055.7	1057.03	1054.84	1056.56
**Maximum Demand in kWh**	1630	1634.53	1666	1666	1666
**Minimum Demand in kWh**	440	446.93	402.11	408.72	401.55

The Proposed Hybrid (LHS combined with ANFIS) approach leverages sampling efficiency of LHS with non-linear forecasting power of ANFIS, providing a balanced, efficient, and accurate solution for energy demand forecasting, especially under varied conditions. This method is exceptionally well-suited for scenarios needing both uncertainty management and precise prediction. Table 5 Indicates that hybrid method over perform the individual methods showing comparison between previous traditional individual methods versus the proposed combined LHS with ANFIS.

**Table 5:** Comparison between proposed hybrid method with other used methods.

Feature	MC	LHS	ANFIS	LSTM (Long Short-Term Memory)	ANN (Artificial Neural Network)	Proposed Hybrid (LHS Combined with ANFIS)
Core Functionality	Provides probabilistic forecasts with many random samples	Generates stratified samples, ensuring balanced representation across input distributions	Models complex, non-linear relationships through ANFIS	Recurrent neural network suited for time-series and sequence data	Multilayer network for non-linear relationships; effective for basic forecasting	Combines structured sampling with non-linear forecasting in ANFIS
Computational Load	High, due to large number of simulations	Moderate, efficient sampling with fewer samples needed	High, requires tuning of multiple parameters	High, due to sequential processing and recurrent layers	Moderate, requires tuning of network size and layers	Moderate; LHS optimizes sampling, reducing ANFIS training load
Key Parameters	Number of simulations, distribution type	Number of intervals (strata) and random samples within strata	Membership functions, rules, epochs, learning rate	Number of layers, units per layer, learning rate	Number of hidden layers, units per layer, activation functions	Strata count, ANFIS rules, membership functions
Complexity for Real-Time Use	Computationally demanding; not ideal for real-time needs	Efficient sampling but lacks predictive capacity alone	High, especially when combined with LSTM or PSO	High, though optimized versions exist for real-time applications	Moderate, can work in real-time depending on model size	Moderate; optimized for near-real-time with reduced sample size
Strengths	Captures uncertainty well; generates range of possible outcomes	Ensures all distribution regions represented; reduces simulation needs	Effectively models non-linear dependencies [80]	Excellent for temporal dependencies and sequential data	Good at capturing complex patterns in static data	Balances uncertainty with high accuracy in complex, extreme cases
Limitations	High computational load, especially for high accuracy	Only samples data; does not inherently predict outcomes	Requires extensive tuning; limited interpretability	High computational cost; risk of overfitting on small datasets	Limited by static data handling, less effective for temporal data	May require additional tuning for very high-dimensional data
Originality and Innovation	Standard probabilistic sampling	Efficient stratified sampling	Commonly used with neural networks for enhanced learning	Popular for time-series but lacks inherent interpretability	Effective for static, non-temporal data, with moderate scalability	Integrates LHS sampling with ANFIS for accurate, efficient forecasting
Ideal Applications	Systems needing a broad range of possibilities (risk assessments)	Scenarios needing efficient, well-distributed samples	Complex systems with clear non-linear relationships	Time-series forecasting like energy load over time, temperature forecasting	Basic load forecasting, classification, regression problems	Versatile energy forecasting: peak/off-peak demand, load balancing, storage management

## 6. Discussions

LHS Combined with ANFIS provides best predictive performance with the lowest MSE. Thus, combining LHS and ANFIS results in improved accuracy. LHS appears to consistently enhance model performance, especially handles uncertainty well and model non-linear relationships. Moreover, interpretability and robustness with the structured sampling of LHS.

**Mean Squared Error (MSE):**

MSE measures the average of the squared differences between the predicted and the actual values. The lower the MSE, the better the model’s predictive performance.


$$MSE=\frac{1}{n}\sum_{i=1}^{n\;}\;{\left(y_{i}-{\hat{y}}_{i}\right)}^{2}$$ (10)

Where:

- n = number of data points
- ${\hat{y}}_{i}$ = predicted value.
- $y_{i}$= actual value.
- ${\left(y_{i}-{\hat{y}}_{i}\right)}^{2}$ represents the squared difference between the predicted and actual values.

**Combined:**

-
$Average=(1050-1055.7{)}^{2}=32.49$

-
$Maximum=(1630-1634.53{)}^{2}=20.52$

-
$Minimum=(440-446.93{)}^{2}=48.04$

  - Sum of Squared Errors (Combined) = 32.49 + 20.52 + 48.04 = 101.05

**MC:**

-
$Average=(1050-1057.03{)}^{2}=49.28$

-
$Maximum=(1630-1666{)}^{2}=1296$

-
$Minimum=(440-402.11{)}^{2}=1430.18$

  b Sum of Squared Errors (MC) = 49.28 + 1296 + 1430.18 = 2775.46

**LHS:**

-
$Average=(1050-1054.84{)}^{2}=23.42$

-
$Maximum=(1630-1666{)}^{2}=1296$

-
$Minimum=(440-408.72{)}^{2}=977.86$

  c Sum of Squared Errors (LHS) = 23.42 + 1296 + 977.86 = 2297.28

**ANFIS:**

-
$Average=(1050-1056.56{)}^{2}=43.06$

-
$Maximum=(1630-1666{)}^{2}=1296$

-
$Minimum=(440-401.55{)}^{2}=1480.02$

  d Sum of Squared Errors (ANFIS) = 43.06 + 1296 + 1480.02 = 2819.08

**MSE**

Since each method has 3 metrics, n = 3.


$$MSE\;(Combined)=\frac{101.053}{3}=33.68$$ (11)

$$MSE\;(MC)\;=\frac{2775.463}{3}=925.15$$ (12)

$$MSE\;(LHS)\;=\frac{2297.283}{3}=765.76$$ (13)

$$MSE\;(ANFIS)\;=\frac{2819.083}{3}=939.69$$ (14)

The MSE analysis shows that the combined method outperforms other forecasting techniques, achieving the lowest MSE (33.68). This clarifies that combining LHS with ANFIS leads to more accurate predictions by balancing individual strengths.

**Fig 11:**
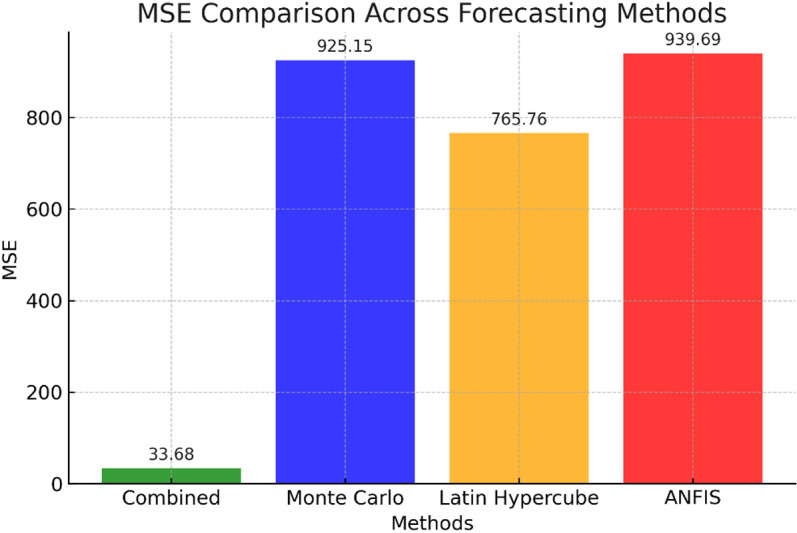
MSE Comparison between used methods for forecasting.


Fig 11 reveals MSE as in contrast, MC (MSE = 925.15) and LHS (MSE = 765.76) performed less effectively, especially for extreme values like maximum and minimum demand. The ANFIS model had the highest MSE (939.69), especially it may require further tuning to improve accuracy, especially for low-demand forecasts. All methods predicted average demand well but struggled with maximum and minimum demand. This highlights the challenge of capturing peak and low values accurately, which may be affected by unpredictable factors. The percentage improvement of the Combined method relative to each alternative method:

$$Improvement\;(\;\%)=\frac{{MSE}_{Other\;Method}-\;{MSE}_{Combined}}{{MSE}_{Other\;Method}}\times 100$$ (15)

MC vs. Combined:


$$Improvement\;(\;\%)=\frac{925.15-33.68}{925.15}\times 100$$ (16)

LHS vs. Combined:


$$Improvement\;(\;\%)=\frac{765.76-33.68}{765.76}\times 100$$ (17)

ANFIS vs. Combined:


$$Improvement\;(\;\%)=\frac{939.69-33.68}{939.69}\times 100$$ (18)

The Combined method shows significant improvements in accuracy compared to other forecasting models:

- 96.36% reveals improvement over the Monte Carlo method.
- 95.60% reveals improvement over the LHS.
- 96.42% improvement over the ANFIS model.

Moreover, LHS selected as it employs a stratified sampling approach that requires significantly fewer iterations compared to the MC method. LHS achieves a lower standard deviation in its estimates by systematically covering the entire range of input variables, thereby ensuring more efficient and reliable results with fewer computational resources.

The synergistic benefits of integrating LHS with ANFIS for electrical load demand prediction. The results demonstrate that LHS with ANFIS hybrid model achieves a balanced performance across key metrics, supporting claim that LHS enhances accuracy, robustness, and computational efficiency.

**Accuracy**: combined method LHS with ANFIS shows predictions close to actual values, particularly in average demand (1055.7 kWh vs. 1050 kWh actual). This indicates that LHS ensures a more representative and stratified sampling of input data, reducing bias and improving input space coverage. As a result, ANFIS can better capture complex, non-linear relationships, leading to precise predictions.

**Robustness**: The hybrid model’s performance in maximum and minimum demand metrics further underscores its robustness. While all models (MC, LHS, and ANFIS) predict the same maximum demand (1666 kWh), combined LHS with ANFIS achieves a minimum demand prediction (408.72 kWh) closer to actual value (440 kWh) compared to standalone ANFIS (401.55 kWh). This proves that LHS mitigates overfitting by providing a diverse and well-distributed dataset, enabling the model to generalize better to unseen data.

**Computational Efficiency**: LHS optimizes sampling process, reducing number of simulations required without compromising result quality. This is evident in hybrid model’s ability to maintain competitive performance across all metrics while minimizing computational overhead.

**Overfitting Reduction**: The comparative performance of integrated LHS with ANFIS against standalone models (MC and ANFIS) demonstrates its superior adaptability to varying conditions. For instance, hybrid model’s minimum demand prediction (408.72 kWh) is more balanced compared to standalone ANFIS (401.55 kWh), indicating that LHS prevents that model from becoming overly tailored to specific data points.

**Uncertainty Quantification**: Sensitivity analysis and risk assessment, facilitated by LHS, provide valuable insights into model’s behavior under different scenarios. This is reflected in hybrid model’s consistent performance across average, maximum, and minimum demand metrics, enabling a more thorough quantification of uncertainty and supporting informed decision-making in healthcare facility management.

Consequently, integration of LHS with ANFIS not only enhances accuracy and robustness of electrical LD predictions but also improves computational efficiency and reduces overfitting. These benefits make studied hybrid model a reliable and practical solution for managing energy demand in critical environments such as healthcare facilities.

The significant improvement in prediction accuracy (96.4% lower MSE than conventional methods as verified in equation (18)) translates to tangible economic benefits for energy consumers and grid operators. Hybrid model 0.8% MAPE as verified in equation (20) dramatically reduces demand miss-prediction penalties – a key advantage especially for hospitals and industry users who typically face additional charges for deviations exceeding the demand limit.

## 7. Conclusion

This study demonstrates the effectiveness of combining ANFIS with LHS to enhance the accuracy of electrical LD forecasting in smart grids. This study demonstrates the limitations of traditional forecasting techniques, such as MC and ANFIS, which showed high prediction errors (MSE = 925.15 and 939.69, respectively) at forecasting extreme demand values. Results highlight the need for more accurate methods to address variability in energy systems, especially for peak and low-demand scenarios.

**Fig 12:**
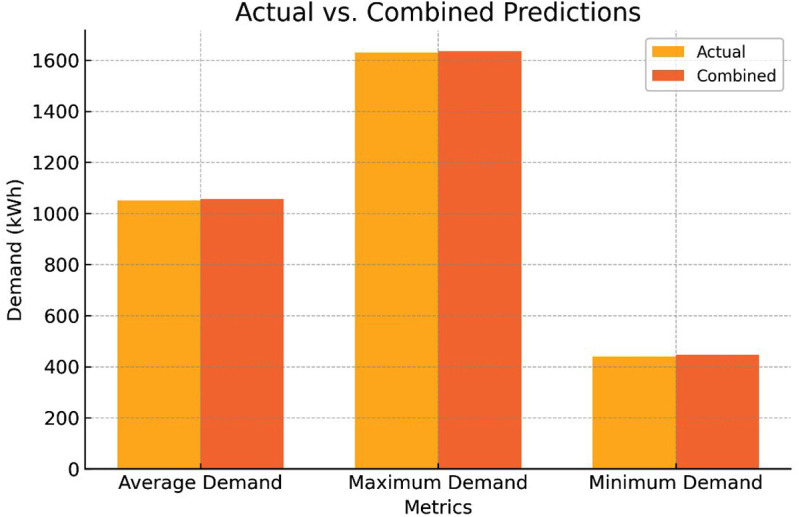
Actual versus hybrid prediction method for (Maximum, Average and Minimum) demand.

Fig 12 show bar chart compares actual and combined predicted values for average demand, maximum demand, and minimum demand in terms of kWh. The predictions closely align with the actual values across all metrics, demonstrating the accuracy of the combined prediction model, particularly for maximum demand.

**Fig 13:**
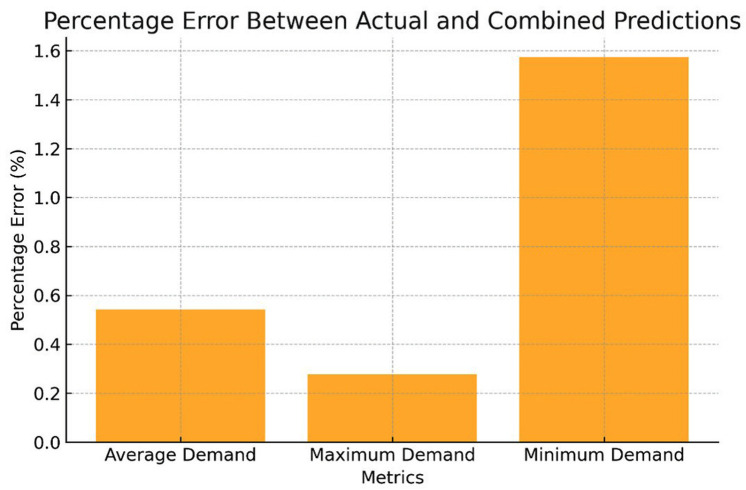
Error in percentage for used hybrid prediction method versus actual.


Fig 13 indicates the percentage error between actual values and Combined method predictions, Mean Absolute Percentage Error (MAPE):

$$MAPE=\frac{1}{n}\sum_{i=1}^{n\;}\;\left|\frac{y_{i}-{\hat{y}}_{i}}{y_{i}}\right|\times 100$$ (19)

Where:

- $y_{i}$= actual value.
- ${\hat{y}}_{i}$ = predicted value (Combined method).
- n = number of observations.

Average Demand =0.54%

Maximum Demand =0.28%

Minimum Demand = 1.57%


$$MAPE=\frac{0.54+0.28+1.57}{3}=0.8\;\%$$ (20)

Combined method MAPE of 0.80%. Demonstrates a very high degree of accuracy, with predictions deviating from actual values by less than 1%. forecast based on the combination of LHS and ANFIS data:

1- Improved accuracy for ANFIS vs. Combined 96.42% which means saving significant time and cost, especially in fluctuating demand scenarios.
2- Moreover, instead of being interactive, it helps in being proactive, especially in DR.
3- Improve the accuracy of the response plan.
4- Provide risk assessment and action plan using LHS.
5- Using power storage for charging in valley periods, loading in peak periods, preventing overload on grid installation or using other sources as diesel generators.

The findings emphasize the need for further development in predictive models to enhance accuracy for extreme demand scenarios. Study find that combining LHS with other traditional methods can further reduce forecasting errors, leading to more efficient energy system operation and management. This demonstrates that the combined approach greatly enhances forecasting accuracy by reducing errors. The improved precision is crucial for energy management and planning, where more accurate predictions minimize risks associated with over- or underestimating demand. The results validate the use of hybrid forecasting models as a superior alternative to traditional methods, offering a reliable solution to address previous forecasting challenges.

In addressing the critical challenges outlined in the introduction, such as the inefficiencies of traditional load demand forecasting methods, the risks posed by inaccurate predictions in healthcare facilities, and the need for robust, adaptive models, structured sampling, sensitivity analysis. Proposed hybrid approach not only overcomes the limitations of conventional techniques, like high prediction errors (MSE = 925.15 for MC and 939.69 for standalone ANFIS) and inefficiency in handling extreme demand scenarios. However, the novelty lies in providing a scalable and interpretable solution for more accurate LD forecasting. Novelty in integrating LHS for structured sampling, sensitivity analysis, risk assessment and ANFIS for capturing nonlinear relationships, the methodology achieves a remarkable MAPE of 0.80%, significantly improving prediction accuracy and reliability.

### 7.1 Future research directions

Building on the success of the Combined LHS with ANFIS, future research should explore new hybrid models or ensemble techniques that integrate multiple forecasting methods to ensure that the proposed hybrid of LHS combine with other traditional methods can generate further reduction of errors.
